# Opposing patterns in eating behaviors following bariatric surgery versus lifestyle-induced weight loss

**DOI:** 10.1371/journal.pone.0346240

**Published:** 2026-04-27

**Authors:** Laura Vuorela, Bram J. Berntzen, Maheswary Muniandy, Tuure Saarinen, Sanna Meriläinen, Vesa Koivukangas, Laura Suojanen, Per-Henrik Groop, Aila Rissanen, Kirsi Virtanen, Anne Juuti, Kirsi H. Pietiläinen, Sini Heinonen

**Affiliations:** 1 Obesity Research Unit, Research Program for Clinical and Molecular Metabolism, Faculty of Medicine, University of Helsinki, Helsinki, Finland; 2 Department of Public Health, Finnish Twin Cohort Study, University of Helsinki, Helsinki, Finland; 3 Institute for Molecular Medicine Finland (FIMM), University of Helsinki, Helsinki, Finland; 4 Department of Gastrointestinal Surgery, Abdominal Center, Helsinki University Hospital and University of Helsinki, Helsinki, Finland; 5 Abdominal Center, Oulu University Hospital, Medical Research Center Oulu, University of Oulu, Oulu, Finland; 6 Department of Nephrology, University of Helsinki and Helsinki University Hospital, Finland; 7 Research Program for Clinical and Molecular Metabolism, Faculty of Medicine, University of Helsinki, Helsinki, Finland; 8 Folkhälsan Institute of Genetics, Folkhälsan Research Center, Biomedicum Helsinki, Finland; 9 Department of Diabetes, Central Clinical School, Monash University, Melbourne, Australia; 10 Department of Medicine, Endocrinology and Clinical Nutrition, Kuopio University Hospital, Kuopio, Finland; 11 Turku PET Center, Turku University Hospital, Turku, Finland; 12 HealthyWeightHub, Endocrinology, Abdominal Center, Helsinki University Hospital and University of Helsinki, Helsinki, Finland; 13 Department of Internal medicine and rehabilitation, Helsinki University Hospital, Helsinki, Finland; University of Rome Tor Vergata: Universita degli Studi di Roma Tor Vergata, ITALY

## Abstract

**Objective:**

This study aimed to investigate 12-month changes in eating behaviors and metabolic outcomes following bariatric surgery or lifestyle-induced weight loss.

**Methods:**

This study is a longitudinal secondary analysis comparing data from two independent prospective cohorts: bariatric surgery (n = 19) and lifestyle-induced weight loss intervention (n = 19). Body weight, body composition (Dual-energy X-ray absorptiometer – DEXA), and metabolic parameters (blood samples, Oral Glucose Tolerance test – OGTT) were measured, and eating behaviors were assessed using validated questionnaires (Three-Factor Eating Questionnaire, Dutch Eating Behavior Questionnaire, Binge-Eating Scale) at baseline, at 5–6 months, and at 12 months after the intervention initiation.

**Results:**

Bariatric surgery produced greater weight loss (surgery −28.1 ± 8.1 kg vs lifestyle −8.9 ± 7.9 kg; p < 0.001) and larger improvements in metabolic markers than lifestyle-induced weight loss. Despite these differences, eating-behavior trajectories diverged. Bariatric surgery was from baseline to 12 months associated with stable or decreased eating restraint, whereas lifestyle-induced weight loss led to an increase in restraint (cognitive restraint: surgery −0.05 ± 0.7 vs lifestyle +6.4 ± 0.9; p < 0.001; restrained eating: surgery −0.7 ± 0.1 vs lifestyle +0.6 ± 0.2; p < 0.001). Both interventions reduced disinhibited eating, binge eating, and external eating. Hunger-related outcomes also improved in both interventions, but with different signatures: surgery was accompanied by reduced hunger perception and cue-reactivity, whereas lifestyle-induced weight loss was characterized by increased reliance on deliberate cognitive/behavioral control strategies. When changes in eating behaviors were analyzed per 1% body weight lost, the opposing pattern in restraint remained significant, and the lifestyle group showed a larger increase in restraint and a greater relative reduction in susceptibility to hunger compared with surgery. Exploratory item-level patterns supported these profiles, suggesting fewer cue-driven triggers to overeat after surgery and greater use of conscious restraint strategies after lifestyle-induced weight loss.

**Conclusions:**

Lifestyle-induced weight loss was associated with increased cognitive restraint, whereas bariatric surgery resulted in stable or decreased restraint. Both interventions decreased hunger sensations, likely through adaptive learning in the lifestyle group and physiological changes after surgery.

## Introduction

Obesity is a chronic, relapsing disease associated with complications such as type 2 diabetes (T2D), cardiovascular disease, and various cancers [1]. Despite treatment advances, achieving and maintaining weight loss remains a major challenge.

Long-term weight loss outcomes differ drastically between intervention methods [2]. Bariatric surgery results in significant and sustained weight loss, reduced cardiovascular risk, remission of T2D, and improved quality of life [3,4]. While lifestyle interventions also yield metabolic benefits, they usually produce less weight loss and are associated with poor long-term maintenance and frequent weight regain [5].

Bariatric surgery and lifestyle-induced weight loss affect physiology differently, influencing food intake regulation [6,7]. Surgery promotes sustained weight loss through mechanisms that enhance satiety, reduce appetite and hunger [8–10], and facilitate psychological changes in eating behavior, such as decreased motivation to eat [9], and reduced desire to eat high-calorie treats [11–14]. Individuals undergoing bariatric surgery also seem to better control their desires [15,16]. In contrast, findings regarding eating behavior following lifestyle-induced weight loss are inconsistent. Studies have reported increases [17,18], no changes [19], or decreases [20–22] in hunger and hunger-driven eating following lifestyle interventions, depending on the approach. Unlike bariatric surgery, lifestyle-induced weight loss often requires more conscious, sustained efforts to restrict food intake and regulate eating behaviors [22,23].

Few studies have directly compared changes in eating behavior following bariatric surgery versus lifestyle-induced weight loss. One comparative study found that surgery reduced hunger, appetite, and the effort needed to stop eating [24]. Even when appetite decreased similarly after both interventions [25], the desire to eat and prospective food consumption declined more after surgery. To date, only two studies have evaluated changes in eating behaviors using validated questionnaires to directly compare bariatric surgery and lifestyle-induced weight loss [26,27]. At 11 weeks, hedonic hunger decreased similarly after a comparable weight loss in both groups, although reductions in food reward across categories were greater following surgery [26]. Reductions in emotional eating, hunger, disinhibition and food reward along with increases in eating restraint and sustained weight loss, were observed up to one year after bariatric surgery, whereas participants in the lifestyle-induced weight loss group experienced weight regain and no long-term improvements in eating behaviors [27].

Therefore, the aim of this study was to identify key differences in eating behavior changes between bariatric surgery and lifestyle-induced weight loss, using validated questionnaires and a one-year follow-up. This study evaluates both interventions using standardized, multidimensional assessments and uniquely adjusts for weight loss to better isolate behavioral changes. Factors underlying these changes are explored through subscale analysis and qualitative evaluation of individual questionnaire items.

## Methods

### Participants

This is a secondary, comparative, longitudinal analysis of two independent prospective cohorts with harmonized assessments. We included adults without type 2 diabetes who had complete eating-behavior questionnaire data available at baseline and at 12-month follow-up.

#### Bariatric surgery cohort.

The parent bariatric surgery cohort (n = 120) has been previously published) [28]. At 12 months, 112 participants remained with complete follow-up (one planned gastric bypass was converted to sleeve gastrectomy, three were lost to follow-up, and four did not return questionnaires). For the present comparative analysis, we selected a subset of 19 participants without diabetes whose baseline body mass index and sex distribution were most comparable to the lifestyle cohort. These participants (14 women/5 men; age 47.0 ± 9.2 years; baseline body mass index >35 kg/m²) underwent Roux-en-Y gastric bypass (n = 7) or one-anastomosis gastric bypass (n = 12). Key exclusions included anemia (hemoglobin <120 g/L), pregnancy/lactation, contraindications to magnetic resonance imaging/spectroscopy, hiatal hernia, reflux esophagitis or Barrett’s esophagus, and other conditions affecting safety or interpretation.

#### Lifestyle cohort.

Nineteen adults with obesity (12 women/ 7 men; age 35.8 ± 7.7 years) completed a lifestyle weight-loss trial, had no concomitant medications, and had complete follow-up [29–31]. Exclusions included smoking, > 5 kg weight change in the prior 3 months, diabetes, endocrine disease, or medications affecting appetite or weight regulation; no participants were lost to follow-up.

Participants were recruited at Helsinki University Central Hospital and Oulu University Hospital (surgery) and the University of Helsinki Obesity Research Unit (lifestyle). Ethics approvals were HUS 1/13/03/02/16 (surgery) and 270/13/03/01/08 (lifestyle); trials were registered at ClinicalTrials.gov (NCT02882685; NCT01312090). All participants gave written informed consent.

### Study design

Both cohorts were followed for 12 months with repeated measures of eating behaviors, anthropometry/body composition, and metabolic outcomes.

#### Surgery timeline.

Baseline was at the start of a 6-week very-low-calorie diet (800–1000 kcal/day), ~ 6 weeks pre-surgery; follow-ups occurred at 6 and 12 months post-surgery. Subsequently, participants received nutritional and exercise counseling adapted to postsurgical needs [28].

#### Lifestyle timeline.

Assessments were at baseline, 5 months, and 12 months. The program began with a 6-week very-low-calorie diet, followed by counseling for a 500–1000 kcal/day deficit with regular dietitian/nurse visits (twice monthly to month 5, then monthly) [29–31].

Although not designed as a head-to-head trial, the similar follow-up structure and identical eating-behavior questionnaires enabled longitudinal comparison of behavioral trajectories between interventions.

### Clinical examinations

Body weight and height were measured in light clothing after an overnight fast, and body mass index (BMI, kg/m^2^) was calculated. Body composition was measured by dual-energy x-ray absorptiometry (DEXA; GE Lunar Prodigy). Fasting plasma glucose was measured following a 12-hour fast using spectrophotometric hexokinase and glucose-6-phosphate dehydrogenase assay (Roche Diagnostics), and insulin resistance estimated using the homeostatic model assessment for insulin resistance (HOMA-IR) index. Fasting plasma total cholesterol, HDL cholesterol, and triglycerides were measured using enzymatic methods (Roche Diagnostics Hitachi, Hitachi Ltd) and LDL cholesterol was calculated using the Friedewald formula.

### Questionnaires

Eating behaviors were assessed using validated questionnaires: (i) the Three-Factor Eating Questionnaire (TFEQ), (ii) the Dutch Eating Behavior Questionnaire (DEBQ), and (iii) the Binge-Eating Scale (BES; Table 1). The TFEQ measured cognitive restraint of eating (conscious restriction of food intake), disinhibited eating (overeating in response to social, emotional, or food-related triggers), and susceptibility to hunger (Table 1) [32]. The TFEQ subscales included: flexible and rigid control [33]; habitual, emotional, and situational susceptibility to disinhibition [34]; and internal and external locus of hunger [34]. The DEBQ assessed restrained, external, and emotional eating [35]. The BES evaluated the severity of binge-eating behavior [36]. Participants completed the Finnish versions of the questionnaires. Responses were scored based on questionnaires’ instructions. In the rare occurrence that participants gave two answers for a single item, the average was recorded. Outcome variables were included if at least 80% of items were completed. In the surgery group, one DEBQ item (Question 22: “Do you have a desire to eat when you are emotionally upset?”), was missing by default and treated as a missing response. Additionally, we conducted a qualitative analysis by selecting individual items per questionnaire that showed more prominent changes (based on *p* values and value shifts) between baseline and 12-month follow-up. We selected ten items from TFEQ and DEBQ, and five from BES, to facilitate a more descriptive discussion.

**Table 1:** Definitions and scoring for eating behavior variables.

	Definition	Scale
**TFEQ**		AU
Cognitive restraint of eating	Conscious effort to restrict food consumption in order to control body weight	0–21
Rigid control	Strict guidelines and an “all or nothing” kind of behavior to restrict food intake	0–7
Flexible control	Subtle approach to limiting food intake with adaptable boundaries	0–7
Disinhibited eating	Overeating or losing control of eating in response to emotional, social, or food property (sight, smell, and taste) triggers	0–16
Situational disinhibition	Overeating triggered by social circumstances or other environmental triggers	0–5
Habitual disinhibition	Repeated patterns of overeating due to frequent environmental triggers	0–5
Emotional disinhibition	Overeating in response to negative emotions	0–3
Susceptibility to hunger	Influence of hunger perception on food consumption	0–14
Internal locus for hunger	Hunger that is regulated by the body’s internal signals	0–6
External locus for hunger	Hunger that is activated by external cues	0–6
**DEBQ**		
External eating	Overeating in response to external cues	1–5
Restrained eating	The intention to restrict eating	1–5
Emotional eating	Eating due to negative feelings	1–5
**BES**		
Binge-eating score	The extent to which one experiences losing control of eating, a preoccupation with food, and eating and related dysphoric feelings Severe binge eating Moderate binge eating No binge eating	0–46 ≥27 18–26 ≤17

Abbreviations: TFEQ, Three-Factor Eating Questionnaire; AU, arbitrary units; DEBQ, Dutch Eating Behavior Questionnaire; BES, Binge-Eating Scale. Based on Berntzen BJ 2021 thesis citation [37].

### Statistical methods

Statistical analyses were conducted using Stata (release 17.0, Stata Corporation, College Station, TX, USA) and R (R Foundation for Statistical Computing, Vienna, Austria). Between- and within-group differences in outcome variables from baseline to 5–6 and 12 months were analyzed using linear mixed-effects models with restricted maximum likelihood estimation. The models included group, time and their interaction as fixed effects, as well as sex, age, baseline BMI, and baseline outcome value to account for baseline differences between groups. Participant identifiers were included as random effects. Residuals were assessed for normality using the Shapiro–Wilk test and by visual inspection of quantile–quantile (QQ) plots and histograms. Variables were log10-transformed if residual distribution violated normality assumptions. Baseline between-group differences were assessed using separate linear mixed models, with group-and-time interaction as fixed and participant id’s as random effects. Chi-square test was used to assess baseline sex differences. To account for differences in weight loss between groups, we investigated behavioral changes relative to weight loss percentage. Linear mixed-effects regression models were used to evaluate changes in standardized eating behaviors per 1% of weight loss from baseline to 12 months, adjusted for sex and age. In this analysis the eating behavior variables were additionally standardized (mean = 0, SD = 1) to facilitate comparisons. Post hoc comparisons were performed using The Tukey HSD test. Individual questionnaire items were compared between baseline and at 12 months using the McNemar symmetry test for dependent variables. All statistical test were two-tailed, and significance was set at *p* < 0.05. Because the study sample size was determined by feasibility, post hoc effect size estimates and power calculations were used to contextualize the detectable between-group differences in eating behavior trajectories. Effect size was quantified as Cohen’s d for the between-group difference in change from baseline to the final follow-up timepoint, corresponding to the group × time interaction estimate from the linear mixed-effects models. Post hoc power was calculated using G*Power with the test family t tests (two-tailed) “Means: Difference between two independent means,” using α = 0.05 and group sample sizes of n = 19 each. Under these conditions, a standardized effect size of approximately Cohen’s d = 0.95 is required to achieve 80% power, indicating that the study was primarily powered to detect large between-group differences in behavioral change. Given the strong conceptual relatedness among eating behaviors and the emphasis on estimation of behavioral trajectories rather than binary hypothesis testing, no formal correction for multiple comparisons was applied. Effect size estimates and corresponding power calculations for all eating behavior outcomes are reported in the Supporting Information to allow interpretation of effect magnitude and uncertainty across behaviors (S1 Table).

## Results

### Greater metabolic improvements following bariatric surgery compared to lifestyle-induced weight loss

**Fig 1:**
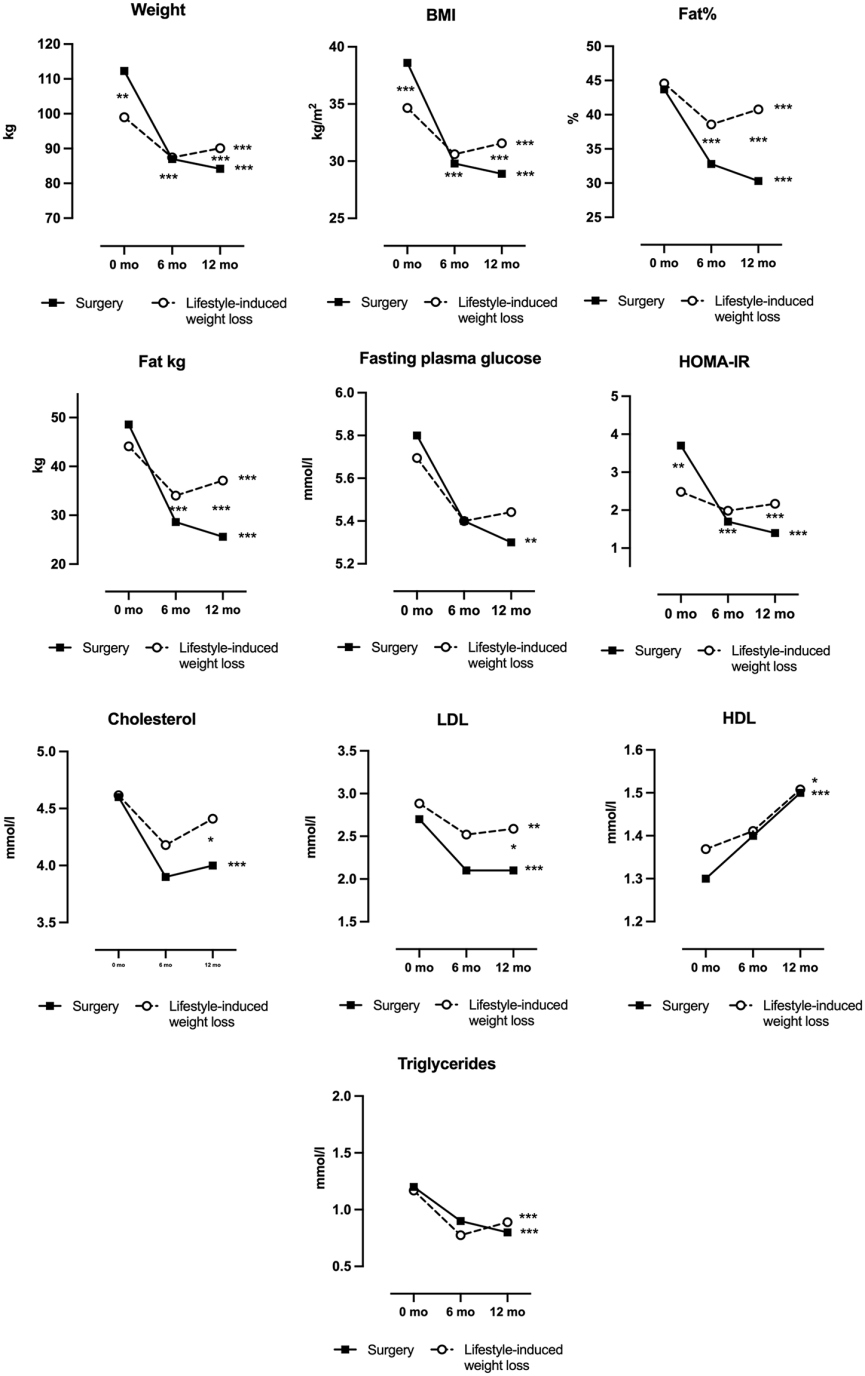
Anthropometric and metabolic characteristics in bariatric surgery- versus lifestyle-induced weight loss interventions at baseline and at 5 months for the dieting group and 6 months for the bariatric surgery group and 12 months. The *p*-values for comparisons between surgery and dieting interventions are shown as asterisks between the surgery and dieting data points at each timepoint. The *p*-values for the within-group change from baseline to 12 months is shown behind the final data point of the dieting and surgery group separately. *p < 0.05,**p < 0.01***, *p* < 0.001. Abbreviations: BMI, body mass index; HOMA-IR, homeostatic model assessment for insulin resistance; LDL, low-density lipoprotein; HDL, high-density lipoprotein.

At baseline, bariatric surgery participants had a higher BMI (38.6 ± 2.5 vs. 34.6 ± 2.7; p < 0.001) as well as higher weight, age, and HOMA-index but lower HbA1c levels compared to the lifestyle-induced weight loss group. No other significant differences were observed between the groups (Fig 1, Table 2, S2 Table). None of the participants had type 2 diabetes. By 5–6 months, the bariatric surgery group showed greater reductions in weight, BMI, body fat, as well as larger improvements in glucose metabolism, including Hba1c and HOMA-index, compared to the lifestyle group. These differences persisted at 12 months, with surgery participants also showing more pronounced improvements in lipid profile by higher decreases in total and LDL-cholesterol levels. At 12 months, the bariatric surgery group had achieved a total weight loss of 28.1 ± 8.1 kg (25.1 ± 7.2%), significantly greater than the 8.9 ± 7.9 kg (9.0 ± 7.4%) lost in the lifestyle-induced weight loss group (p < 0.001). Although both groups had a similar baseline body fat percentage (~44%), the surgery group had a 10.5% lower body fat percentage than the lifestyle group at 12 months (Fig 1, Table 2, S2 Table).

**Table 2:** Between-group comparisons of weight loss and metabolism: bariatric surgery versus lifestyle-induced weight loss intervention at baseline, 5/6 months, and 12 months.

Surgery vs. Lifestyle								
	Baseline		Baseline vs. 5/6 months		5/6 vs. 12		Baseline vs. 12 months	
Variable	Effect size [95% CI]	p-value	Effect size [95% CI]	p-value	Effect size [95% CI]	p-value	Effect size [95% CI]	p-value
Age (years)	−11.3 [−14.4, −8.2]	**<0.001**						
Female sex(%)	74 vs 63%	0.887						
Height (cm)	−1.8 [−4.9, 1.3]	0.244						
Weight (kg)	−13.3 [−21.6, 5.0]	**0.002**	13.7 [9.7, 17.7]	**<0.001**	5.5 [1.5, 9.5]	**0.007**	19.2 [15.2, 23.2]	**<0.001**
BMI (kg/m2)	−6.1 [−6.3, −1.7]	**<0.001**	4.7 [3.3, 6.1]	**<0.001**	1.9 [0.5, 3.3]	**0.007**	6.7 [5.3, 8.1]	**<0.001**
Fat (%)	0.8 [−4.2, 5.9]	0.745	4.9 [2.3, 7.5]	**<0.001**	4.6 [2.0, 7.2]	**0.001**	9.5 [6.9, 12.1]	**<0.001**
Fat (kg)	−4.5 [10.1, 1.2]	0.121	9.9 [6.4, 13.3]	**<0.001**	6.2 [2.7, 9.7]	**0.001**	16.0 [12.6, 19.5]	**<0.001**
fP-glucose (mmol/l)	−0.1 [−0.5, 0.3]	0.585	0.1 [−0.3, 0.5]	0.543	0.1 [−0.3, 0.5]	0.603	0.2 [−0.2, 0.7]	0.253
Hba1c (mmol/mol)	2.5 [−0.1, 5.0]	0.055	2.8 [1.3, 4.2]	**<0.001**				
HOMA-index	−1.8 [−2.0, −0.4]	**0.009**	0.2 [0.1, 0.4]	**<0.001**	0.1 [−0.1, 0.2]	0.349	0.3 [0.2, 0.4]	**<0.001**
Cholesterol (mmol/l)	0.1 [−0.4, 0.5)	0.814	0.2 [−0.1, 0.5]	0.263	0.2 [−0.2, 0.5]	0.285	0.4 [0.0, 0.7]	**0.027**
HDL (mmol/l)	0.03 [−0.2, 0.2]	0.712	0.0 [0.0, 0.0]	0.815	0.0 [−0.1, 0.1]	0.294	−0.0 [−0.1, 0.0]	0.196
LDL (mmol/l)	0.1 [−0.3, 0.6]	0.520	0.2 [−0.1, 0.5]	0.137	0.1 [−0.4, 0.2]	0.517	0.3 [0.0, 0.6]	**0.032**
Triglycerides (mmol/l)	−0.05 [−0.3, 0.2]	0.703	−0.1 [−0.2, 0.0]	0.179	0.1 [0.0, 0.2]	**0.029**	0.0 [−0.1, 0.1]	0.398
Weight loss (kg)			−13.7 [−17.7, −9.7]	**<0.001**	−5.5 [−9.5, −1.5]	**0.007**	−19.2 [−23.2, −15.2]	**<0.001**
Weight loss (%)			−10.8 [−14.4, −7.2]	**<0.001**	−5.3 [−9.0, −1.7]	**0.004**	−16.1 [−19.8, −12.5]	**<0.001**

Linear mixed models were used to assess longitudinal timepoint differences. Models were adjusted for sex, age, baseline BMI and baseline value of the outcome variable. Baseline between-group differences were assessed using separate unadjusted linear mixed models. Chi-square test was used to assess baseline sex differences. Significant values are shown in bold. Abbreviations: BMI, body mass index; HOMA-IR, homeostatic model assessment for insulin resistance; LDL, low-density lipoprotein; HDL, high-density lipoprotein.

### Increased cognitive restraint following lifestyle-induced weight loss, reduced disinhibited eating following bariatric surgery, and decreased susceptibility to hunger in TFEQ following both interventions

**Fig 2:**
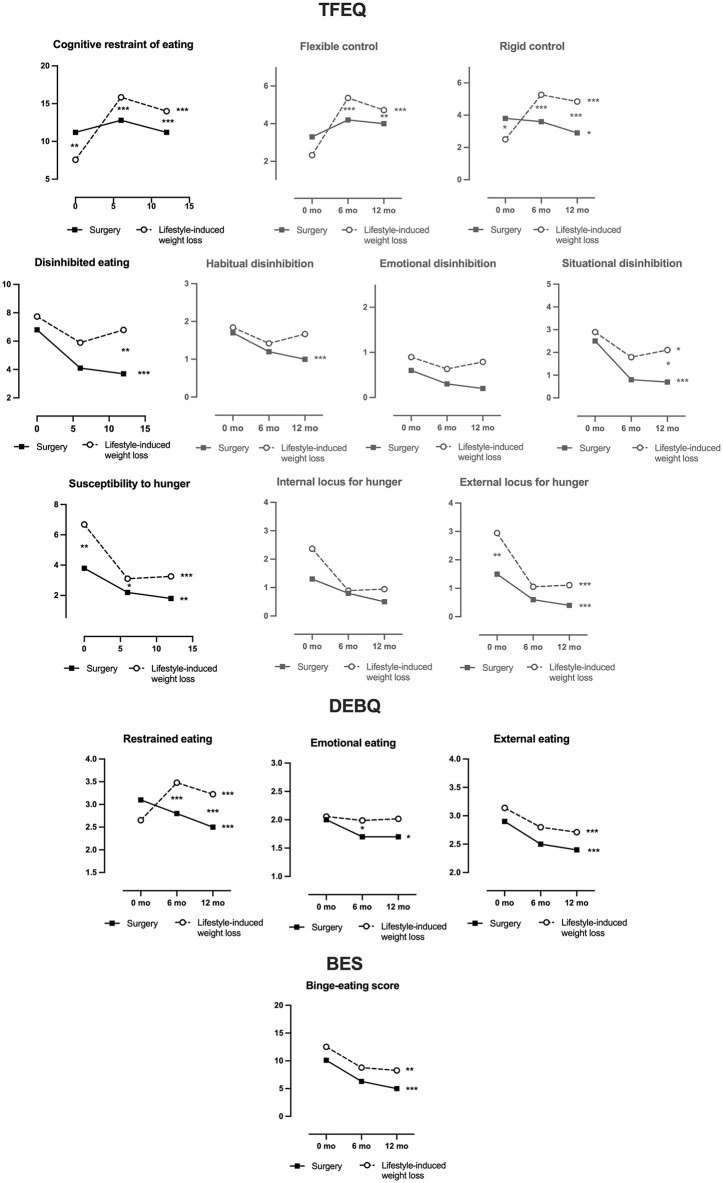
Eating behaviors in bariatric surgery- versus lifestyle-induced weight loss interventions at baseline and at 5 months for the dieting group and 6 months for the bariatric surgery group and 12 months. The primary variables in the graphs are colored black and the subscale graphs are colored grey. The *p*-values for the comparisons between surgery and dieting are shown as asterisks between the data points at each timepoint. The *p*-values for the within-group change from baseline to 12 months are shown behind the final data point of the dieting and surgery group separately. *p < 0.05, **p < 0.01, ***p < 0.001. Abbreviations: TFEQ, Three-Factor Eating Questionnaire; DEBQ, Dutch Eating Behavior Questionnaire; BES, Binge-Eating Scale.

First, we assessed eating behaviors using the TFEQ. At baseline, cognitive restraint of eating was higher in bariatric surgery than lifestyle-induced weight loss participants (Fig 2, Table 3, S3 Table). During weight loss, cognitive restraint significantly increased at both 6 and 12 months in the lifestyle-induced weight loss group but remained unchanged in bariatric surgery participants, resulting in significant between-group differences at both timepoints. Disinhibited eating, also similar at baseline, decreased in both groups, though the reduction was more pronounced in the bariatric surgery group over the 12-month period. At baseline, both lifestyle-induced group reported higher levels of susceptibility to hunger. However, participants in the lifestyle-induced weight loss group showed a greater reduction during the first 6 months compared with those in the bariatric surgery group. By 12 months, susceptibility to hunger had significantly declined in both groups, with no significant between-group differences (Fig 2, Table 3, S3 Table).

**Table 3:** Between-group comparisons of eating behaviors: bariatric surgery versus lifestyle-induced weight loss intervention at baseline, 5/6 months, and 12 months.

Surgery vs. Lifestyle								
	Baseline		Baseline vs. 5/6 months		5/6 vs. 12		Baseline vs. 12 months	
Variable	Effect size [95% CI]	p-value	Effect size [95% CI]	p-value	Effect size [95% CI]	p-value	Effect size [95% CI]	p-value
**Cognitive restraint of eating**	−3.6 [−6.4, 0.9]	**0.009**	7.0 [4.5, 9.5]	**<0.001**	−0.5 [−3.0, 2.0]	0.682	6.5 [4.0, 8.9]	**<0.001**
**Flexible control**	−0.9 [−2.2, 0.2]	0.113	2.2 [1.0, 3.4]	**<0.001**	−0.6 [−1.8, 0.7]	0.373	1.7 [0.4, 2.9]	**0.008**
**Rigid control**	−1.3 [−2.4, −0.2]	**0.017**	3.1 [2.1, 4.1]	**<0.001**	0.2 [−0.8, 1.2]	0.699	3.3 [2.3, 4.3]	**<0.001**
**Disinhibited eating**	0.9 [−1.0, 2.8]	0.506	0.1 [0.0, 0.2]	0.198	0.1 [0.0, 0.2]	0.066	0.2 [0.1, 0.3]	**0.002**
**Habitual disinhibition**	0.1 [−0.6, 0.8]	0.765	0.1 [−0.5, 0.7]	0.641	0.4 [−0.2, 1.0]	0.204	0.5 [−0.1, 1.1]	0.078
**Emotional disinhibition**	0.3 [−0.3, 0.9]	0.310	0.1 [−0.5, 0.6]	0.815	0.2 [−0.3, 0.7]	0.453	0.3 [−0.3, 0.8]	0.321
**Situational disinhibition**	0.4 [−0.4, 1.3]	0.337	0.5 [−0.4, 1.5]	0.266	0.5 [−0.5, 1.4]	0.354	1.0 [0.0, 2.0]	**0.04**
**Susceptibility to hunger**	2.8 [1.2, 4.5]	**0.002**	−2.0 [−3.7, −0.3]	**0.024**	0.6 [−1.2, 2.3]	0.516	−1.4 [−3.1, 0.3]	0.105
**Internal locus for hunger**	1.1 [0.3, 1.8]	0.337	−0.1 [−0.4, 0.1]	0.289	0.0 [−0.3, 0.3]	0.935	−0.1 [−0.4, 0.2]	0.413
**External locus for hunger**	1.4 [0.5, 2.2]	**0.002**	−0.9 [−1.8, 0.1]	0.065	0.4 [−0.6, 1.3]	0.448	−0.5 [−1.5, 0.4]	0.277
**Restrained eating**	−0.5 [−0.9, 0.1]	0.816	1.2 [0.8, 1.6]	**<0.001**	0.1 [−0.3, 0.5]	0.72	1.2 [0.8, 1.6]	**<0.001**
**Emotional eating**	0.03 [−0.5, 0.5]	0.904	0.4 [0.0, 0.8]	**0.029**	−0.2 [−0.5, 0.2]	0.383	0.2 [−0.1, 0.6]	0.19
**External eating**	0.3 [−0.3, 0.6]	0.077	0.1 [−0.2, 0.3]	0.572	0.0 [−0.2, 0.2]	0.964	0.1 [−0.2, 0.3]	0.539
**Binge-eating score**	2.5 [−1.0, 5.9]	0.159	0.0 [−3.2, 3.2]	0.988	1.1 [−2.1, 4.3]	0.496	1.1 [−2.1, 4.3]	0.502

Linear mixed models were used to assess longitudinal between-group differences. Models were adjusted for sex, age, baseline BMI and baseline value of the outcome variable. Baseline between-group differences were assessed using separate unadjusted models. Significant values are shown in bold.

Subscale analysis revealed that flexible (adaptable) and rigid (strict) control, components of cognitive restraint, increased in the lifestyle-induced weight loss group, resulting in significant differences from the bariatric surgery group at 5–6 and 12 months. In contrast, flexible control remained stable after surgery, while rigid control decreased over the 12-month period. Among disinhibition subscales, habitual disinhibition decreased only in the bariatric surgery group, whereas emotional disinhibition remained unchanged in both groups. Situational disinhibition decreased in both groups, with a significantly greater reduction in the surgery group. Regarding hunger subscales, sensitivity to external hunger cues significantly decreased in both groups, while the internal hunger signals remained unchanged over 12 months. Observed standardized effect sizes varied across eating behavior outcomes, resulting in substantial variability in achieved power (S1 Table); consequently, null findings for outcomes with small to moderate effect sizes should be interpreted cautiously.

### Decreased restrained, emotional, and external eating in DEBQ following bariatric surgery, and increased restraint and decreased external eating following lifestyle-induced weight loss

Next, we examined eating behaviors using the DEBQ questionnaire. At baseline, restrained eating did not differ between the bariatric surgery and lifestyle-induced weight loss groups (Fig 2, Table 3, S3 Table). Over time, restrained eating decreased following bariatric surgery and increased after lifestyle-induced weight loss, resulting in a significant between-group difference at both 5–6 and 12 months. Emotional eating triggered by negative feelings declined significantly in the bariatric surgery group, whereas no changes were observed in the lifestyle-induced weight loss group, resulting in a significant between-group difference at 5–6 months. External eating driven by sensory cues (smell, taste, or sight of food) decreased similarly in both groups over 12 months.

### Absence of binge-eating disorder, with decreased binge eating behaviors during weight loss in both groups

Additionally, we assessed the severity of the binge eating patterns using the BES. No binge-eating disorder was reported in either group at any timepoint. BES scores were similar at baseline and decreased over 12 months in both groups, with no significant differences in the extent of reduction between the bariatric surgery and lifestyle-induced weight loss groups at 5–6 and 12 months (Fig 2, Table 3, S3 Table).

### Higher cognitive restraint and greater reductions in hunger susceptibility following lifestyle-induced weight loss than with bariatric surgery after accounting for weight loss

**Fig 3:**
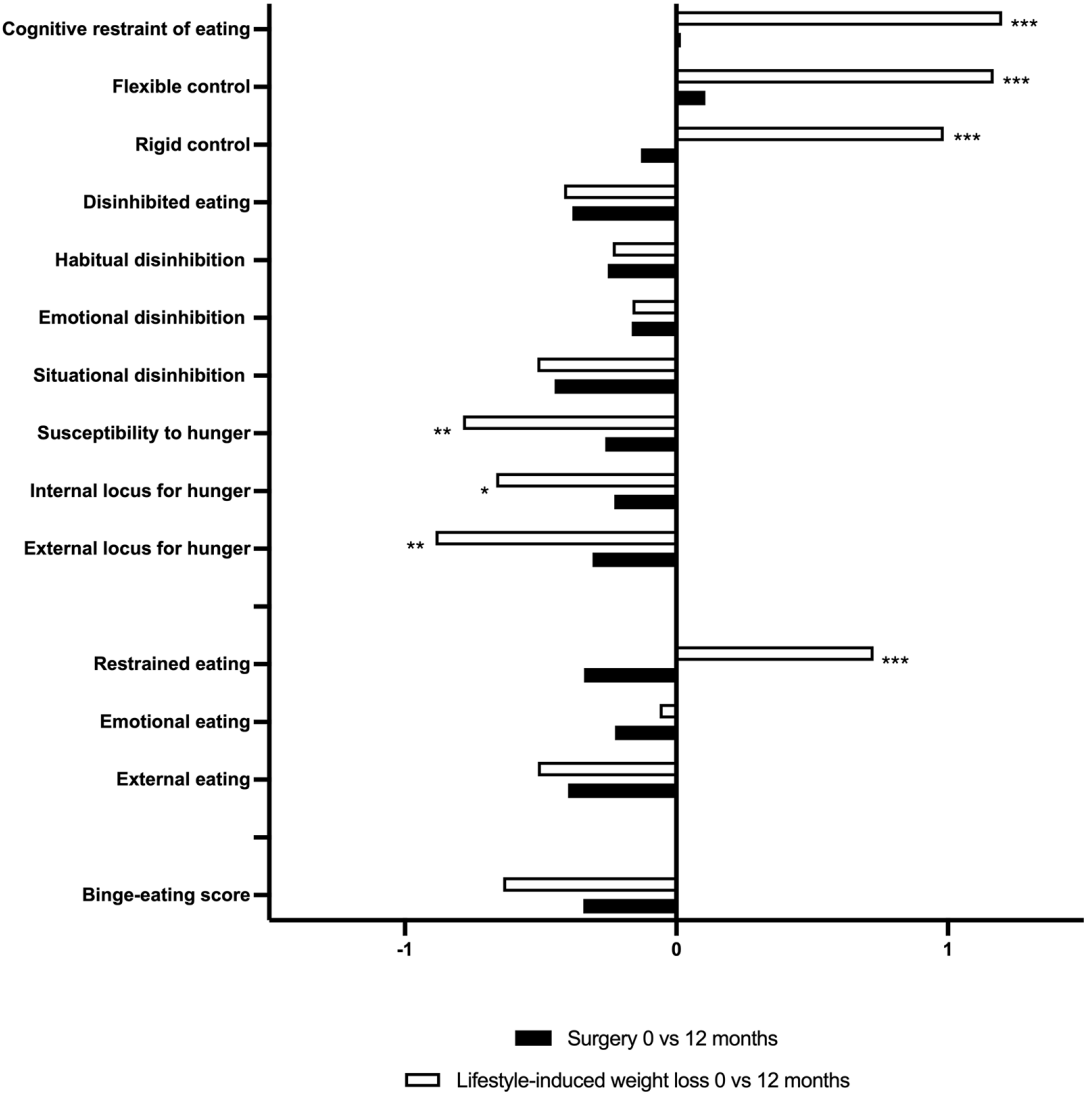
Change in standardized eating behaviors per percentage of weight loss between 0 and 12 months in the bariatric surgery- versus lifestyle-induced weight loss interventions. *p < 0.05, **p < 0.01, ***p < 0.001. Abbreviations: TFEQ, Three-Factor Eating Questionnaire; DEBQ, Dutch Eating Behavior Questionnaire; BES, Binge-Eating scale.

During follow-up, participants in the bariatric surgery group lost more weight than those in the lifestyle-induced weight loss group. To account for this difference, changes in eating behaviors were analyzed relative to each 1% of weight lost (Fig 3, Table 4). All variables were also standardized (i.e., z-scores) to enable direct comparison across scales.

**Table 4:** Standardized eating behaviors in the bariatric surgery- and lifestyle-induced weight loss intervention groups per percentage of weight loss.

	Surgery 0 vs 12 months		Lifestyle 0 vs 12 months		Surgery vs Lifestyle
Variable	Standardized beta coefficient	*p*-value	Standardized beta coefficient	*p*-value	*p*-value
Cognitive restraint of eating	0.02	0.822	1.20	**<0.001**	**<0.001**
Flexible control	0.11	0.170	1.17	**<0.001**	**<0.001**
Rigid control	−0.13	0.099	0.99	**<0.001**	**<0.001**
Disinhibited eating	−0.38	**<0.001**	−0.41	**<0.001**	0.808
Habitual disinhibition	−0.25	**0.001**	−0.23	0.095	0.904
Emotional disinhibition	−0.17	**0.020**	−0.16	0.233	0.984
Situational disinhibition	−0.45	**<0.001**	−0.51	**0.001**	0.727
Susceptibility to hunger	−0.26	**0.002**	−0.79	**<0.001**	**0.004**
Internal locus for hunger	−0.23	**0.025**	−0.66	**<0.001**	**0.040**
External locus for hunger	−0.31	**<0.001**	−0.89	**<0.001**	**0.002**
Restrained eating	−0.34	**<0.001**	0.73	**<0.001**	**<0.001**
Emotional eating	−0.23	**<0.001**	−0.06	0.603	0.222
External eating	−0.40	**<0.001**	−0.51	**<0.001**	0.222
Binge-eating score	−0.34	**<0.001**	−0.64	**<0.001**	0.054

Regression analysis, using linear mixed-effects models, was used to analyze the change in standardized eating behaviors per 1% of weight loss between baseline and the final follow-up visit. The models were adjusted for sex and age. The Tukey HSD test was used as the post hoc analysis to compare surgery versus dieting. Significant values are shown in bold.

These analyses showed opposite behavioral profiles in the two interventions. Over 12 months, the largest standardized eating behavioral changes per percentage of weight loss in the bariatric surgery group were decreases in the main scale for disinhibited eating (β = −0.38), the subscale for situational disinhibition (β = −0.45), and external eating (β = −0.40). In contrast, the most pronounced changes in the lifestyle-induced weight loss group were increases in the main scale for cognitive restraint of eating (β = +1.20), the subscales for flexible control (β = +1.17) and rigid control (β = +0.99).

Compared to the surgery group, the lifestyle-induced weight loss group showed significantly greater increases in restrained eating (DEBQ) and cognitive restraint (TFEQ), including both flexible and rigid control subscales, indicating an opposing pattern between the groups, even after adjusting for weight loss. Moreover, the lifestyle-induced weight loss group demonstrated a significantly greater reduction in susceptibility to hunger, including both internal and external hunger subscales, compared with the surgery group. For other eating behavioral traits, the same amount of weight loss from both interventions was associated with a similar degree of change between the groups.

### Individual questions revealed decreased triggers to overeat following bariatric surgery and increased restraint behaviors following lifestyle-induced weight loss

To better understand the nature of the reported eating-behavior changes, we conducted an exploratory, descriptive examination of individual questionnaire items from the TFEQ, DEBQ, and BES over the 12-month follow-up (S4–S9 Tables). Given the limited sample size, these item-level observations should be interpreted as hypothesis-generating rather than inferential.

In TFEQ, descriptive patterns suggested a decrease in disinhibited eating following bariatric surgery, and an increase in cognitive restraint of eating with lifestyle-induced weight loss (S4 Table, S7 Table). After surgery, participants tended to report less overeating triggered by appealing food or by seeing others overeat, as well as fewer difficulties leaving food on their plate or stopping eating. In addition, it seemed that strict control over food intake became less necessary, as conscious dieting efforts occurred less frequently. In contrast, participants in the lifestyle-induced weight loss group appeared to show greater reliance on conscious restraint strategies, such as consciously eating less than desired or adjusting intake in response to weight fluctuations. However, both groups described adopting small behavioral changes to regulate intake (e.g., taking smaller portions or skipping dessert). Another notable similarity between the groups was related to hunger. After bariatric surgery, diminished hunger was a commonly reported theme, making dietary adherence less challenging and reducing the urge to eat spontaneously. Following lifestyle-induced weight loss, the response to the sight of delicious food appeared less pronounced, and participants demonstrated improved hunger control, finding it easier to stop eating before finishing their meals.

Exploratory item-level trends in the DEBQ (S5 Table, S8 Table) showed reduced external and restrained eating post-surgery. Interestingly, participants in the lifestyle-induced weight loss group appeared to combine reduced external eating with increases in restrained eating. After bariatric surgery, the taste, smell, sight, or availability of delicious food seemed to have a diminished impact on eating. In the lifestyle-induced weight loss group, participants reported fewer temptations to buy food, less overeating of tasty foods, and greater resistance to eating treats. Weight loss following bariatric surgery was also associated with reduced avoidance of eating to control weight and fewer compensatory behaviors after overeating. In contrast, participants in the lifestyle-induced weight loss group reported increased restrained eating, reflected in more frequent refusal of foods or drinks due to weight concerns, stricter monitoring of food intake and eating less than desired.

BES item patterns post-surgery (S6 Table) indicated a reduction in polarized eating behaviors (i.e., overeating followed by fasting), fewer preoccupations with controlling urges to eat, and decreased self-consciousness regarding body weight or size. Similarly, participants in the lifestyle-induced weight loss group (S9 Table) also reported less polarized eating, with a reduced tendency to overeat until uncomfortably full or to eat impulsively without experiencing physical hunger.

These exploratory patterns provide qualitative context for the broader behavioral changes observed at the scale and subscale level but should not be interpreted as independent statistical findings.

## Discussion

This study compared one-year changes in eating behaviors and metabolic outcomes after bariatric surgery and lifestyle-induced weight loss. Despite larger weight loss and metabolic improvements after surgery, lifestyle-induced weight loss was characterized by a greater increase in eating restraint and control, and this divergence persisted after analyzing changes per 1% body weight lost in each cohort. Both interventions were associated with reductions in overeating-related traits, reactivity to external food cues, and susceptibility to hunger, but the pattern of change differed. After surgery, improvements in hunger and cue-driven eating occurred alongside stable or reduced restraint, consistent with a more automatic shift in appetite and eating regulation. In contrast, lifestyle-induced weight loss combined reductions in overeating and external eating with a marked rise in conscious, effortful restraint strategies. These findings suggest that early eating-behavior adaptation after weight loss is intervention-specific and may have implications for long-term weight maintenance.

Most changes in eating behaviors occurred within the first 5–6 months, corresponding to the period of greatest weight loss and most frequent monitoring. Over time, however, some behavioral changes may diminish or reverse, particularly in the lifestyle-induced weight loss group, which showed a trend toward weight regain after six months. Supporting this, a previous study reported that positive changes in eating behavior, such as decreases in emotional eating, hunger, disinhibition, and food reward and increases in dietary restraint, persisted for up to a year after bariatric surgery but not after lifestyle-induced weight loss, where weight regain was observed [27]. These findings suggest that lifestyle-induced weight loss may primarily lead to short-term behavioral adaptations.

Notably, we observed opposing trajectories in restrained eating (DEBQ) following bariatric surgery and lifestyle-induced weight loss. Specifically, eating restraint increased with lifestyle-induced weight loss but decreased after surgery. A similar pattern emerged in the TFEQ subscale for rigid control, whereas flexible control increased only in the lifestyle-induced weight loss group and remained unchanged after bariatric surgery. Some studies have reported unchanged [15,38–40] or even increased [41] eating restraint after bariatric surgery, partly contradicting our findings. In contrast, many studies have shown increases in eating restraint following lifestyle-induced weight loss [21,23,42,43]. Interestingly, one comparative study reported increased dietary restraint one year after intervention only in the bariatric surgery group but not after lifestyle-induced weight loss [27], differing from our results. However, our findings align with studies showing reductions in desire to eat and prospective food consumption following bariatric surgery [25], as well as reduced preference for highly palatable foods, observed through functional magnetic resonance imaging [14,44]. These changes may reduce the need for conscious restraint, consistent with our observation that favorable shifts in eating restraint and weight occur post-surgery without strict effort. In contrast, participants in the lifestyle group appeared to require more active behavioral strategies to maintain dietary control. This divergence may reflect physiology: satiety hormones [e.g., glucagon like peptide 1 [45,46], peptide YY [45,47,48], and cholecystokinine [45,47]] increase after bariatric surgery but remain unchanged or decrease after lifestyle-induced weight loss. This may help to explain why lifestyle-induced weight loss necessitates more deliberate control over eating, whereas post-surgical changes appear more biologically driven.

Similar changes in eating patterns following bariatric surgery and lifestyle-induced weight loss included reduced susceptibility to hunger, less loss of control overeating, and decreased responsiveness to external food cues. However, the decrease in disinhibition (TFEQ) appeared more pronounced in the bariatric surgery group at 12 months, while the lifestyle-induced weight loss group showed only a transient decrease at 5 months. Reductions in externally driven eating and overeating following surgery have also been reported previously [15,39–41], whereas reductions following lifestyle interventions are typically short-term [21,22], consistent with the patterns seen in our data. Supporting this, one study demonstrated a decrease in disinhibition after bariatric surgery, but not after lifestyle-induced weight loss at one year [27]. Evidence suggests that bariatric surgery may naturally reduce cravings [16], whereas lifestyle-induced weight loss may require active strategies to manage overeating [43,49]. Two studies directly comparing surgical and lifestyle-induced weight loss reported reductions in hunger, increases in fullness [25] and decreased hedonic hunger [26,27] in both groups, in line with our results showing diminished hunger after both interventions. Another comparative study found that surgery participants experienced less hunger and greater satiety, while the lifestyle-induced weight loss group reported the opposite [50]. Several additional studies have consistently reported diminished hunger following bariatric surgery [8–10]. However, evidence for hunger changes following lifestyle-induced weight loss is mixed, with some studies reporting decreased [20–22] and others increased [17,18] feelings of hunger. These discrepancies may be explained by differences in measurement tools. Specifically, the TFEQ captures behavioral consequences of hunger and how hunger drives food consumption, providing insight into patterns participants can learn to regulate. Studies using the TFEQ have shown decreases in hunger related eating behavior following lifestyle-induced weight loss, consistent with our findings [21,22]. In contrast, studies using visual analog scale primarily assess immediate perceptions of hunger, which have been shown to increase following lifestyle-induced weight loss [17,18]. It is therefore plausible that, while subjective perception of hunger may increase, individuals may simultaneously learn to respond to these cues with greater restraint.

To explore behavioral changes relative to weight loss in both bariatric surgery and lifestyle-induced weight loss groups, we standardized eating behavior scores and scaled them according to percentage of weight loss. This analysis confirmed an opposing pattern in eating restraint: a decrease after surgery and an increase after lifestyle-induced weight loss. Thus, even after accounting for weight loss, participants in the lifestyle-induced weight loss group appeared to require more conscious effort to limit food intake, whereas restriction became less necessary after surgery. Another notable finding related to hunger, which decreased more per percentage of weight loss in the lifestyle-induced weight loss group than in the surgery group. Although hunger declined in both groups, the lifestyle-induced group experienced less overall weight loss, which may have contributed to the relatively greater reduction in hunger per percentage of lost weight. These findings align with previous studies reporting mixed results on hunger following lifestyle-induced weight loss [17,18,20–22]. This may partly reflect the development of learned behaviors aimed at regulating hunger and managing food intake, though further research is needed.

The changes in eating patterns following bariatric surgery may stem from neurohormonal and physiological changes, including hypothalamic signaling, shifts in gut hormone and bile acid secretion, and changes in the gut microbiota [6]. In contrast, lifestyle-induced weight loss may involve endocrine adaptations in gastrointestinal hormones that resist continued weight loss and may promote weight regain [7]. One comparative study confirmed that gastrointestinal hormone profiles shifted more favorably to regulate hunger just 10 weeks following bariatric surgery compared with lifestyle intervention [25]. These physiological benefits likely contribute to reduced hunger and diminished temptation to overeat after surgery, whereas individuals undergoing lifestyle intervention must actively restrict eating and overcome biological mechanisms that oppose further weight loss.

To better understand which behavioral elements might have influenced the observed changes, we conducted an exploratory, descriptive examination of the qualitative characteristics of eating behaviors by identifying the individual questionnaire items that showed the greatest change over time in each intervention group. Bariatric surgery participants seemed to become less vulnerable to internal signals and environmental triggers for (over)eating and more often reported a decreased need for cognitive control, as conscious dieting efforts diminished. In contrast, participants in the lifestyle-induced weight loss group seemed to adopt learned behaviors that required sustained attention to limit food intake, such as eating less than desired and refusing foods or drinks due to weight concerns. Although both groups reported a diminished susceptibility to hunger and external eating cues, the nature of these changes differed. Bariatric surgery participants more often reported reduced hunger itself (e.g., they were no longer constantly hungry and needed less conscious dieting as hunger decreased), while the lifestyle-induced weight loss group participants rather answered strategies to manage persistent hunger (e.g., eating less than desired, skipping dessert, taking smaller portions). Similarly, bariatric surgery participants reported responding less to sensory food cues (e.g., taste, smell, and sight of food, or social situations), whereas lifestyle-induced weight loss group participants seemed to adopt situational coping strategies to manage such triggers (e.g., inhibiting to buy from local food outlets and resisting delicious foods).

Both groups reported behaviors like skipping dessert when satiated or reducing portion size, but possibly for different reasons. After surgery, these behaviors might have stemmed from physical limitations and discomfort, including nausea and vomiting [24]. In contrast, lifestyle-induced weight loss participants likely adopted these behaviors to control food intake [24]. A 1994 study by Greenstein [24] found that bariatric surgery participants primarily stopped eating to avoid vomiting, whereas lifestyle-induced weight loss participants stopped for appearance or health. This distinction supports our findings: bariatric surgery may promote physical satiety, while lifestyle-induced weight loss can rely on psychological motivation to restrict intake. Thus, the quality and source of behavioral changes after bariatric surgery may contribute to more sustainable long-term outcomes.

Strengths of this study include the standardized clinical setting, with assessments performed in the same research unit by the same researchers, study nurses, and dietitians, as well as comparable follow-up duration and similar sample sizes between groups. Both studies used intensive, structured follow-up with high visit attendance; accordingly, we had complete 12-month follow-up for the participants included in the present analysis in both the lifestyle and surgical groups. We identified key changes in eating behavior one year after both interventions, providing a foundation for future research into physiological mechanisms underlying these behavioral adaptations. However, our study also has limitations. First, eating behaviors were assessed using self-reported questionnaires, which may introduce reporting bias. Nonetheless, these instruments are widely used and validated. Second, the relatively small sample size limits the generalizability of our findings. Third, weight-loss trajectories differed between interventions: weight loss plateaued in the lifestyle group by 6 months, whereas it continued through 12 months after surgery. Because these trajectories may both influence and be influenced by eating behaviors, longer follow-up with larger samples will be important to clarify the temporal relationships and their implications for weight maintenance. Additionally, the groups differed in certain baseline metabolic and behavioral characteristics, as they were not originally designed for direct comparison. To address this, we adjusted analyses for age, sex, baseline BMI and baseline outcome values. We also standardized analyses by percentage of weight loss to enable more equitable comparisons. The use of individual questionnaire items in this study sheds light to the interpretation of the data but should regarded as exploratory and hypothesis-generating rather than confirmatory. Lastly, because weight, metabolic outcomes, and eating behaviors were measured at the same time points, we cannot interfere causality between behavioral and physiological changes.

## Conclusion

Bariatric surgery was associated with a natural reduction or stabilization of eating restraint, whereas lifestyle-induced weight loss required increased cognitive control and conscious restriction of food intake. Importantly, this opposing pattern remained significant even after adjusting for weight loss. While both interventions reduced susceptibility to hunger, overeating, and external eating, bariatric surgery led to a greater reduction in overeating. Interestingly, when adjusted for percentage weight loss, lifestyle-induced weight loss was associated with a greater relative decrease in hunger. However, the nature of this change differed: bariatric surgery appeared to diminish physical hunger and reactivity to external food cues, whereas lifestyle-induced weight loss prompted the adoption of compensatory behaviors to cope with sustained hunger and external eating triggers. These findings suggest that maintaining weight loss and favorable eating behaviors may be more cognitively demanding after lifestyle-induced weight loss due to the increased need for ongoing self-regulation. Further research is needed to identify the physiological mechanisms underlying these behavioral adaptations.

## Supporting information

S1 Table Effect sizes (Cohen’s d) and statistical power for eating behavior variables. Effect size was quantified as Cohen’s d for the between-group difference in change from baseline to the final follow-up timepoint, corresponding to the group × time interaction estimate from the linear mixed-effects models. Post hoc power was calculated using G*Power with the test family t tests.
(DOCX)

S2 Table Weight loss and metabolism following bariatric surgery and lifestyle-induced weight loss at baseline, 5/6 months, and 12 months. Linear mixed models were used to assess timepoint differences. Models were adjusted for sex, age, baseline BMI and baseline value of the outcome variable. Values are reported as mean ± standard deviation (SD). Significant values are shown in bold. Abbreviations: SD, standard deviation; BMI, body mass index; HOMA-IR, homeostatic model assessment for insulin resistance; LDL, low-density lipoprotein; HDL, high-density lipoprotein.
(DOCX)

S3 Table Eating behaviors following bariatric surgery and lifestyle-induced weight loss at baseline, 5/6 months, and 12 months. Linear mixed models were used to assess timepoint differences. Models were adjusted for sex, age, baseline BMI and baseline value of the outcome variable. Values are reported as mean ± standard deviation (SD). Significant values are shown in bold.
(DOCX)

S4 Table Most changed individual questions from TFEQ between baseline and 12 months in the bariatric surgery induced weight loss group. Abbreviations: Q, question; T1, timepoint 1 (0 months); T3, timepoint 3 (12 months). For comparisons, we used McNemar’s test of symmetry for dependent variables and considered *p* < 0.05 statistically significant. Significant values are shown in bold.
(DOCX)

S5 Table Most changed individual questions from DEBQ between baseline and 12 months in the bariatric surgery induced weight loss group. Abbreviations: Q, question; T1, timepoint 1 (0 months); T3, timepoint 3 (12 months). For comparisons, we used McNemar’s test of symmetry for dependent variables and considered *p* < 0.05 statistically significant. Significant values are shown in bold.
(DOCX)

S6 Table Most changed individual questions from BES between baseline and 12 months in the bariatric surgery induced weight loss group. Abbreviations: Q, question; T1, timepoint 1 (0 months); T3, timepoint 3 (12 months). For comparisons, we used McNemar’s test of symmetry for dependent variables and considered *p* < 0.05 statistically significant. Significant values are shown in bold.
(DOCX)

S7 Table Most changed individual questions from TFEQ between baseline and 12 months in the lifestyle-induced weight loss group. Abbreviations: Q, question; T1, timepoint 1 (0 months); T3, timepoint 3 (12 months). For comparisons, we used McNemar’s test of symmetry for dependent variables and considered *p* < 0.05 statistically significant. Significant values are shown in bold.
(DOCX)

S8 Table Most changed individual questions from DEBQ between baseline and 12 months in the lifestyle-induced weight loss group. Abbreviations: Q, question; T1, timepoint 1 (0 months); T3, timepoint 3 (12 months). For comparisons, we used McNemar’s test of symmetry for dependent variables and considered *p* < 0.05 statistically significant. Significant values are shown in bold.
(DOCX)

S9 Table Most changed individual questions from BES between baseline and 12 months in the lifestyle-induced weight loss group. Abbreviations: Q, question; T1, timepoint 1 (0 months); T3, timepoint 3 (12 months). For comparisons, we used McNemar’s test of symmetry for dependent variables and considered *p* < 0.05 statistically significant. Significant values are shown in bold.
(DOCX)
