# Supplementary material for: Opposing patterns in eating behaviors following bariatric surgery versus lifestyle-induced weight loss
Source: PLoS One. 2026 Apr 27;21(4):e0346240. doi: 10.1371/journal.pone.0346240 (PMC13119899; doi:10.1371/journal.pone.0346240)
Supplement: S1 Table — Effect size was quantified as Cohen’s d for the between-group difference in change from baseline to the final follow-up timepoint, corresponding to the group × time interaction estimate from the linear mixed-effects models. Post hoc power was calculated using G*Power with the test family t tests. (DOCX) [file pone.0346240.s001.docx]

**Supplementary Table 1. Effect sizes (Cohen’s d) and statistical power for eating behavior variables.**

| **Variable** | **Cohen's d** | **Power** |
| --- | --- | --- |
| **Cognitive restraint of eating** | 2.36 | 1.0 |
| **Flexible control** | 1.25 | 0.96 |
| **Rigid control** | 3.00 | 1.0 |
| **Disinhibited eating** | 1.45 | 0.99 |
| **Habitual disinhibition** | 0.82 | 0.69 |
| **Emotional disinhibition** | 0.46 | 0.28 |
| **Situational disinhibition** | 0.94 | 0.81 |
| **Susceptibility to hunger** | -0.74 | 0.61 |
| **Internal locus for hunger** | -0.61 | 0.45 |
| **External locus for hunger** | -0.51 | 0.33 |
| **Restrained eating** | 2.81 | 1.0 |
| **Emotional eating** | 0.61 | 0.44 |
| **External eating** | 0.29 | 0.14 |
| **Binge-eating score** | 0.31 | 0.15 |

Effect size was quantified as Cohen’s d for the between-group difference in change from baseline to the final follow-up timepoint, corresponding to the group × time interaction estimate from the linear mixed-effects models. Post hoc power was calculated using G*Power with the test family t tests.
