# Supplementary material for: Opposing patterns in eating behaviors following bariatric surgery versus lifestyle-induced weight loss
Source: PLoS One. 2026 Apr 27;21(4):e0346240. doi: 10.1371/journal.pone.0346240 (PMC13119899; doi:10.1371/journal.pone.0346240)
Supplement: S2 Table — Linear mixed models were used to assess timepoint differences. Models were adjusted for sex, age, baseline BMI and baseline value of the outcome variable. Values are reported as mean ± standard deviation (SD). Significant values are shown in bold. Abbreviations: SD, standard deviation; BMI, body mass index; HOMA-IR, homeostatic model assessment for insulin resistance; LDL, low-density lipoprotein; HDL, high-density lipoprotein. (DOCX) [file pone.0346240.s002.docx]

**Supplementary Table 2.** **Weight loss and metabolism following bariatric surgery and lifestyle-induced weight loss at baseline, 5/6 months, and 12 months.**

|  | **Surgery** | | | | | | | | | | **Lifestyle** | | | | | | | | | | | |
| --- | --- | --- | --- | --- | --- | --- | --- | --- | --- | --- | --- | --- | --- | --- | --- | --- | --- | --- | --- | --- | --- | --- |
|  | **O months** | | **6 months** | | **12 months** | | **Baseline vs 6** | **6 vs 12** | **Baseline vs 12** | **O months** | | | **5 months** | | **12 months** | | **Baseline vs 5** | **5 vs 12** | **Baseline vs 12** | |  |  |
| **Variable** | **n (%)** | **Mean ± SD** | **n (%)** | **Mean ± SD** | **n (%)** | **Mean ± SD** | **p-value** | | | **n (%)** | | **Mean ± SD** | **n (%)** | **Mean ± SD** | **n (%)** | **Mean ± SD** | **p-value** | | |  |  |  |
| Age (years) | 19 (100) | 47.0±9.2 |  |  |  |  |  |  |  | 19 (100) | | 35.8±7.7 |  |  |  |  |  |  |  |  |  |  |
| Female sex (n (%)) | 19 (100) | 14 (74) |  |  |  |  |  |  |  | | 19 (100) | 12 (63%) |  |  |  |  |  |  |  | | |  |
| Height (cm) | 19 (100) | 170.5±7.0 |  |  |  |  |  |  |  | | 19 (100) | 168.7±9.7 |  |  |  |  |  |  |  | | |  |
| Weight (kg) | 19 (100) | 112.3±10.9 | 19 (100) | 87.0±11.8 | 19 (100) | 84.2±11.9 | **<0.001** | **0.047** | **<0.001** | | 19 (100) | 99.0±14.0 | 19 (100) | 87.4±14.1 | 19 (100) | 90.1±15.0 | **<0.001** | 0.065 | **<0.001** | | |  |
| BMI (kg/m2) | 19 (100) | 38.6±2.5 | 19 (100) | 29.8±2.5 | 19 (100) | 28.9±2.7 | **<0.001** | 0.055 | **<0.001** | | 19 (100) | 34.6±2.7 | 19 (100) | 30.6±3.4 | 19 (100) | 31.6±3.9 | **<0.001** | 0.058 | **<0.001** | | |  |
| Fat (%) | 19 (100) | 43.7±7.1 | 19 (100) | 32.8±7.2 | 18 (95) | 30.3±7.9 | **<0.001** | **0.012** | **<0.001** | | 19 (100) | 44.6±7.2 | 19 (100) | 38.6±9.1 | 19 (100) | 40.8±9.2 | **<0.001** | **0.018** | **<0.001** | | |  |
| Fat (kg) | 19 (100) | 48.6±8.6 | 19 (100) | 28.6±7.7 | 18 (95) | 25.6±8.4 | **<0.001** | **0.015** | **<0.001** | | 19 (100) | 44.1±7.9 | 19 (100) | 34.0±9.6 | 19 (100) | 37.1±11.0 | **<0.001** | **0.013** | **<0.001** | | |  |
| fP-glucose (mmol/l) | 18 (95) | 5.8±0.7 | 16 (84) | 5.4±0.9 | 17 (89) | 5.3±0.4 | **0.006** | 0.659 | **0.001** | | 19 (100) | 5.7±0.5 | 19 (100) | 5.4±0.5 | 19 (100) | 5.4±0.7 | **0.043** | 0.773 | 0.083 | | |  |
| Hba1c (mmol/mol) | 19 (100) | 35.0±5.0 | 18 (95) | 31.7±4.6 | 19 (100) | 32.3±4.4 | **<0.001** | 0.309 | **<0.001** | | 19 (100) | 37.5±3.2 | 19 (100) | 37.0±3.0 |  |  | 0.380 |  |  | | |  |
| HOMA-IR | 18 (95) | 3.7±1.5 | 16 (84) | 1.7±0.8 | 17 (89) | 1.4±0.4 | **<0.001** | 0.415 | **<0.001** | | 19 (100) | 2.5±1.3 | 19 (100) | 2.0±1.2 | 19 (100) | 2.2±1.4 | **0.017** | 0.619 | 0.058 | | |  |
| Cholesterol (mmol/l) | 19 (100) | 4.6±0.9 | 18 (95) | 3.9±0.8 | 19 (100) | 4.0±0.8 | **<0.001** | 0.706 | **<0.001** | | 19 (100) | 4.6±0.7 | 19 (100) | 4.2±0.7 | 19 (100) | 4.4±0.7 | **<0.001** | 0.056 | 0.091 | | |  |
| HLD (mmol/l) | 19 (100) | 1.3±0.3 | 18 (95) | 1.4±0.3 | 19 (100) | 1.5±0.2 | 0.164 | **0.003** | **<0.001** | | 19 (100) | 1.4±0.3 | 19 (100) | 1.4±0.3 | 18 (95) | 1.5±0.3 | 0.281 | 0.136 | **0.011** | | |  |
| LDL (mmol/l) | 19 (100) | 2.7±0.7 | 18 (95) | 2.1±0.6 | 19 (100) | 2.1±0.8 | **<0.001** | 0.722 | **<0.001** | | 19 (100) | 2.9±0.6 | 19 (100) | 2.5±0.6 | 18 (95) | 2.6±0.6 | **<0.001** | 0.574 | **0.003** | | |  |
| Triglycerides (mmol/l) | 19 (100) | 1.2±0.5 | 18 (95) | 0.9±0.2 | 19 (100) | 0.8±0.3 | **0.001** | 0.176 | **<0.001** | | 19 (100) | 1.2±0.5 | 19 (100) | 0.8±0.3 | 19 (100) | 0.9±0.4 | **<0.001** | 0.083 | **<0.001** | | |  |
| Weight loss (kg) |  |  | 19 (100) | 25.3±7.1 | 19 (100) | 28.1±8.1 | **<0.001** | **0.047** | **<0.001** | |  |  | 19 (100) | 11.6±5.6 | 19 (100) | 8.9±7.9 | **<0.001** | 0.065 | **<0.001** | | |  |
| Weight loss (%) |  |  | 19 (100) | 22.6±6.2 | 19 (100) | 25.1±7.2 | **<0.001** | 0.052 | **<0.001** | |  |  | 19 (100) | 11.8±5.3 | 19 (100) | 9.0±7.4 | **<0.001** | **0.035** | **<0.001** | | |  |

Linear mixed models were used to assess timepoint differences. Models were adjusted for sex, age, baseline BMI and baseline value of the outcome variable. Values are reported as mean ± standard deviation (SD). Significant values are shown in bold. Abbreviations: SD, standard deviation; BMI, body mass index; HOMA-IR, homeostatic model assessment for insulin resistance; LDL, low-density lipoprotein; HDL, high-density lipoprotein.
