# Supplementary material for: Opposing patterns in eating behaviors following bariatric surgery versus lifestyle-induced weight loss
Source: PLoS One. 2026 Apr 27;21(4):e0346240. doi: 10.1371/journal.pone.0346240 (PMC13119899; doi:10.1371/journal.pone.0346240)
Supplement: S3 Table — Linear mixed models were used to assess timepoint differences. Models were adjusted for sex, age, baseline BMI and baseline value of the outcome variable. Values are reported as mean ± standard deviation (SD). Significant values are shown in bold. (DOCX) [file pone.0346240.s003.docx]

**Supplementary Table 3. Eating behaviors following bariatric surgery and lifestyle-induced weight loss at baseline, 5/6 months, and 12 months.**

|  | **Surgery** | | | | | | | | | **Lifestyle** | | | | | | | | |
| --- | --- | --- | --- | --- | --- | --- | --- | --- | --- | --- | --- | --- | --- | --- | --- | --- | --- | --- |
|  | **O months** | | **6 months** | | **12 months** | | **Baseline vs. 6** | **6 vs. 12** | **Baseline vs. 12** | **O months** | | **5 months** | | **12 months** | | **Baseline vs. 5** | **5 vs. 12** | **Baseline vs. 12** |
| **Variable** | **n (%)** | **Mean±SD** | **n (%)** | **Mean±SD** | **n (%)** | **Mean±SD** | **p-value** | | | **n (%)** | **Mean±SD** | **n (%)** | **Mean±SD** | **n (%)** | **Mean±SD** | **p-value** | | |
| **Cognitive restraint of eating** | 19 (100) | 11.2±4.6 | 17 (89) | 12.8±4.3 | 19 (100) | 11.2±4.8 | 0.169 | 0.152 | 0.953 | 19 (100) | 7.6±4.3 | 19 (100) | 15.8±3.5 | 19 (100) | 14.0±4.2 | **<0.001** | **0.038** | **<0.001** |
| **Flexible control** | 19 (100) | 3.3±2.0 | 18 (95) | 4.2±2.1 | 19 (100) | 4.0±1.9 | 0.057 | 0.732 | 0.113 | 18 (95) | 2.3±1.6 | 19(100) | 5.4±1.5 | 18 (95) | 4.7±2.2 | **<0.001** | 0.115 | **<0.001** |
| **Rigid control** | 19 (100) | 3.8±1.8 | 17 (89) | 3.6±1.8 | 19 (100) | 2.9±1.8 | 0.400 | 0.146 | **0.018** | 18 (95) | 2.5±1.6 | 19 (100) | 5.3±1.5 | 19 (100) | 4.8±1.8 | **<0.001** | 0.361 | **<0.001** |
| **Disinhibited eating** | 19 (100) | 6.8±2.7 | 18 (95) | 4.1±2.1 | 19 (100) | 3.7±1.9 | **<0.001** | 0.519 | **<0.001** | 19 (100) | 7.7±3.5 | 19 (100) | 5.9±3.8 | 19 (100) | 6.8±3.4 | **<0.001** | **0.049** | 0.101 |
| **Habitual disinhibition** | 19 (100) | 1.7±1.0 | 17 (89) | 1.2±0.9 | 19 (100) | 1.0±0.7 | **0.010** | 0.424 | **<0.001** | 19 (100) | 1.8±1.4 | 19 (100) | 1.4±1.3 | 18 (95) | 1.7±1.1 | **0.046** | 0.318 | 0.335 |
| **Emotional disinhibition** | 19 (100) | 0.6±1.0 | 18 (95) | 0.3±0.8 | 19 (100) | 0.2±0.5 | 0.088 | 0.822 | 0.050 | 19 (100) | 0.9±1.2 | 19 (100) | 0.6±1.1 | 19 (100) | 0.8±1.2 | 0.161 | 0.400 | 0.575 |
| **Situational disinhibition** | 19 (100) | 2.5±1.5 | 18 (95) | 0.8±1.2 | 19 (100) | 0.7±1.2 | **<0.001** | 0.691 | **<0.001** | 19 (100) | 2.9±1.2 | 19 (100) | 1.8±1.6 | 19 (100) | 2.1±1.4 | **0.001** | 0.359 | **0.022** |
| **Susceptibility to hunger** | 19 (100) | 3.8±2.9 | 18 (95) | 2.2±2.3 | 19 (100) | 1.8±2.0 | **0.012** | 0.510 | **0.001** | 19 (100) | 6.7±2.7 | 19 (100) | 3.1±3.0 | 19 (100) | 3.3±2.7 | **<0.001** | 0.799 | **<0.001** |
| **Internal locus for hunger** | 19 (100) | 1.3±1.5 | 18 (95) | 0.8±0.9 | 19 (100) | 0.5±0.9 | 0.239 | 0.251 | 0.790 | 19 (100) | 2.4±1.4 | 19 (100) | 0.9±1.2 | 19 (100) | 0.9±1.3 | **0.003** | 0.114 | 0.301 |
| **External locus for hunger** | 19 (100) | 1.5±1.3 | 17 (89) | 0.6±1.1 | 19 (100) | 0.4±1.0 | **0.003** | 0.649 | **<0.001** | 18 (95) | 2.9±1.7 | 19 (100) | 1.1±1.6 | 18 (95) | 1.1±1.2 | **<0.001** | 0.536 | **<0.001** |
| **Restrained eating** | 19 (100) | 3.1±0.7 | 17 (89) | 2.8±0.7 | 19 (100) | 2.5±0.7 | **0.026** | **0.026** | **<0.001** | 19 (100) | 2.7±0.6 | 19 (100) | 3.5±0.4 | 19 (100) | 3.2±0.6 | **<0.001** | 0.074 | **<0.001** |
| **Emotional eating** | 19 (100) | 2.0±0.9 | 18 (95) | 1.7±0.7 | 19 (100) | 1.7±0.6 | **0.002** | 0.521 | **0.011** | 19 (100) | 2.1±0.8 | 18 (95) | 2.0±0.9 | 18 (95) | 2.0±0.8 | 0.950 | 0.554 | 0.506 |
| **External eating** | 19 (100) | 2.9±0.4 | 18 (95) | 2.5±0.5 | 19 (100) | 2.4±0.5 | **<0.001** | 0.273 | **<0.001** | 18 (95) | 3.1±0.4 | 19 (100) | 2.8±0.4 | 19 (100) | 2.7±0.5 | **<0.001** | 0.306 | **<0.001** |
| **Binge-eating score** | 19 (100) | 10.1±5.5 | 18 (95) | 6.3±4.8 | 19 (100) | 5.0±4.8 | **0.001** | 0.249 | **<0.001** | 19 (100) | 12.5±5.6 | 19 (100) | 8.8±6.1 | 18 (95) | 8.3±5.6 | **0.001** | 0.850 | **0.001** |

Linear mixed models were used to assess timepoint differences. Models were adjusted for sex, age, baseline BMI and baseline value of the outcome variable. Values are reported as mean ± standard deviation (SD). Significant values are shown in bold.
