# Supplementary material for: Opposing patterns in eating behaviors following bariatric surgery versus lifestyle-induced weight loss
Source: PLoS One. 2026 Apr 27;21(4):e0346240. doi: 10.1371/journal.pone.0346240 (PMC13119899; doi:10.1371/journal.pone.0346240)
Supplement: S4 Table — Abbreviations: Q, question; T1, timepoint 1 (0 months); T3, timepoint 3 (12 months). For comparisons, we used McNemar’s test of symmetry for dependent variables and considered p < 0.05 statistically significant. Significant values are shown in bold. (DOCX) [file pone.0346240.s004.docx]

**Supplementary Table 4a. Most changed individual questions from TFEQ between baseline and 12 months in the bariatric surgery induced weight loss group.**

| **Surgery** | **Three Factor Eating Questionnaire** |  |  |  |
| --- | --- | --- | --- | --- |
| Question |  | Behavioral trait | Δmean (T3-T1) | Symmetry test p-value |
| Q7 | Sometimes things just taste so good that I keep on eating even when I am no longer hungry. | Disinhibited eating | -0.5789474 | **0.003** |
| Q13 | When I am with someone who is overeating, I usually overeat too. | Disinhibited eating | -0.42 | **0.008** |
| Q47 | How frequently do you skip dessert because you are no longer hungry? | Susceptibility to hunger | -0.37 | **0.016** |
| Q34 | I am always hungry enough to eat at any time. | Susceptibility to hunger | -0.32 | **0.031** |
| Q37 | How often are you dieting in a conscious effort to control your weight? | Cognitive restraint of eating | -0.32 | **0.031** |
| Q6 | I deliberately take small helpings as a means of controlling my weight. | Cognitive restraint of eating | 0.37 | **0.039** |
| Q8 | Since I am often hungry, I sometimes wish that while I am eating, an expert would tell me that I have had enough or that I can have something more to eat. | Susceptibility to hunger | -0.26 | 0.063 |
| Q5 | Dieting is so hard for me because I just get too hungry. | Cognitive restraint of eating | -0.32 | 0.070 |
| Q16 | It is not difficult for me to leave something on my plate. | Disinhibited eating | -0.32 | 0.11 |
| Q15 | Sometimes when I start eating, I just can’t seem to stop. | Disinhibited eating | -0.26 | 0.13 |
| Q19 | Being with someone who is eating often makes me hungry enough to eat also. | Susceptibility to hunger | -0.26 | 0.13 |

Abbreviations: Q, question; T1, timepoint 1 (0 months); T3, timepoint 3 (12 months).

For comparisons, we used McNemar’s test of symmetry for dependent variables, and considered *p* < 0.05 statistically significant. Significant values are shown in bold.
