# Supplementary material for: Opposing patterns in eating behaviors following bariatric surgery versus lifestyle-induced weight loss
Source: PLoS One. 2026 Apr 27;21(4):e0346240. doi: 10.1371/journal.pone.0346240 (PMC13119899; doi:10.1371/journal.pone.0346240)
Supplement: S5 Table — Abbreviations: Q, question; T1, timepoint 1 (0 months); T3, timepoint 3 (12 months). For comparisons, we used McNemar’s test of symmetry for dependent variables and considered p < 0.05 statistically significant. Significant values are shown in bold. (DOCX) [file pone.0346240.s005.docx]

**Supplementary Table 4b. Most changed individual questions from DEBQ between baseline and 12 months in the bariatric surgery induced weight loss group.**

| **Surgery** | **Dutch Eating Behavior Questionnaire** |  |  |  |
| --- | --- | --- | --- | --- |
| Question |  | Behavioral trait | Δmean (T3-T1) | Symmetry test p-value |
| Q24 | If food tastes good to you, do you eat more than usual? | External eating | -1.21 | **0.001** |
| Q9 | How often in the evening do you try not to eat because you are watching your weight? | Restrained eating | -1.00 | **0.008** |
| Q8 | How often do you try not to eat between meals because you are watching your weight? | Restrained eating | -1.05 | **0.009** |
| Q3 | How often do you refuse food or drink offered because you are concemed about your weight? | Restrained eating | -0.89 | **0.030** |
| Q25 | If food smells and looks good, do you eat more than usual? | External eating | -0.84 | **0.031** |
| Q27 | If you have something delicious to eat, do you eat it straight away? | External eating | -0.53 | **0.031** |
| Q1 | If you have put on weight, do you eat less than you usually do? | Restrained eating | -0.47 | 0.078 |
| Q28 | If you walk past the baker do you have the desire to buy something delicious? | External eating | -0.53 | 0.13 |
| Q21 | Do you feel a desire to eat when you are disappointed? | Emotional eating | -0.47 | 0.13 |
| Q14 | Do you have a desire to eat when you are feeling lonely? | Emotional eating | -0.37 | 0.13 |

Abbreviations: Q, question; T1, timepoint 1 (0 months); T3, timepoint 3 (12 months).

For comparisons, we used McNemar’s test of symmetry for dependent variables, and considered *p* < 0.05 statistically significant. Significant values are shown in bold.
