# Supplementary material for: Opposing patterns in eating behaviors following bariatric surgery versus lifestyle-induced weight loss
Source: PLoS One. 2026 Apr 27;21(4):e0346240. doi: 10.1371/journal.pone.0346240 (PMC13119899; doi:10.1371/journal.pone.0346240)
Supplement: S6 Table — Abbreviations: Q, question; T1, timepoint 1 (0 months); T3, timepoint 3 (12 months). For comparisons, we used McNemar’s test of symmetry for dependent variables and considered p < 0.05 statistically significant. Significant values are shown in bold. (DOCX) [file pone.0346240.s006.docx]

**Supplementary Table 4c. Most changed individual questions from BES between baseline and 12 months in the bariatric surgery induced weight loss group.**

| **Surgery** | **Binge-Eating Scale** |  |  |
| --- | --- | --- | --- |
| Question |  | Δmean (T3-T1) | Symmetry test p-value |
| Q9 | 1. My level of calorie intake does not go up very high or go down very low on a regular basis.  2. Sometimes after I overeat, I will try to reduce my caloric intake to almost nothing to compensate for the excess calories I’ve eaten.  3. I have a regular habit of overeating during the night. It seems that my routine is not to be hungry in the morning but overeat in the evening.  4. In my adult years, I have had week-long periods where I practically starve myself. This follows periods when I overeat. It seems I live a life of either “feast or famine.” | -0.84 | **0.008** |
| Q6 | 1. I don’t feel any guilt or self-hate after I overeat.  2. After I overeat, occasionally I feel guilt or self-hate.  3. Almost all the time I experience strong guilt or self-hate after I overeat. | -0.58 | **0.008** |
| Q3 | 1. I feel capable to control my eating urges when I want to. 2. I feel like I have failed to control my eating more than the average person.  3. I feel utterly helpless when it comes to feeling in control of my eating urges.  4. Because I feel so helpless about controlling my eating I have become very desperate about trying to get in control. | -0.37 | **0.016** |
| Q14 | 1. I don’t think much about trying to control unwanted eating urges.  2. At least some of the time, I feel my thoughts are pre-occupied with trying to control my eating urges.  3. I feel that frequently I spend much time thinking about how much I ate or about trying not to eat anymore.  4. It seems to me that most of my waking hours are pre-occupied by thoughts about eating or not eating. I feel like I’m constantly struggling not to eat | -0.44 | **0.031** |
| Q1 | 1. I don’t feel self-conscious about my weight or body size when I’m with others.  2. I feel concerned about how I look to others, but it normally does not make me feel disappointed with myself.  3. I do get self-conscious about my appearance and weight which makes me feel disappointed in myself.  4. I feel very self-conscious about my weight and frequently, I feel intense shame and disgust for myself. I try to avoid social contacts because of my self- consciousness. | -0.32 | **0.031** |

Abbreviations: Q, question; T1, timepoint 1 (0 months); T3, timepoint 3 (12 months).

For comparisons, we used McNemar’s test of symmetry for dependent variables, and considered *p* < 0.05 statistically significant. Significant values are shown in bold.
