# Supplementary material for: Opposing patterns in eating behaviors following bariatric surgery versus lifestyle-induced weight loss
Source: PLoS One. 2026 Apr 27;21(4):e0346240. doi: 10.1371/journal.pone.0346240 (PMC13119899; doi:10.1371/journal.pone.0346240)
Supplement: S7 Table — Abbreviations: Q, question; T1, timepoint 1 (0 months); T3, timepoint 3 (12 months). For comparisons, we used McNemar’s test of symmetry for dependent variables and considered p < 0.05 statistically significant. Significant values are shown in bold. (DOCX) [file pone.0346240.s007.docx]

**Supplementary Table 4d. Most changed individual questions from TFEQ between baseline and 12 months in the lifestyle-induced weight loss group.**

| **Lifestyle** | **Three Factor Eating Questionnaire** | | | |
| --- | --- | --- | --- | --- |
| Question |  | Behavioral trait | Δmean (T3-T1) | Symmetry test p-value |
| Q48 | How likely are you to consciously eat less than you want? | Cognitive restraint of eating | +0.61 | **0.001** |
| Q6 | I deliberately take small helpings as a means of controlling my weight. | Cognitive restraint of eating | +0.58 | **0.001** |
| Q38 | Would a weight fluctuation of 5 lbs affect the way you live your life? | Cognitive restraint of eating | +0.44 | **0.008** |
| Q22 | When I see a real delicacy, I often get so hungry that I have to eat right away. | Susceptibility to hunger | -0.42 | **0.008** |
| Q5 | Dieting is so hard for me because I just get too hungry. | Cognitive restraint of eating | -0.39 | **0.016** |
| Q15 | Sometimes when I start eating, I just can’t seem to stop. | Disinhibited eating | -0.39 | **0.016** |
| Q47 | How frequently do you skip dessert because you are no longer hungry? | Susceptibility to hunger | -0.39 | **0.016** |
| Q40 | Do your feelings of guilt about overeating help you to control your food intake? | Cognitive restraint of eating | +0.39 | **0.016** |
| Q14 | I have a pretty good idea of the number of calories in common food | Cognitive restraint of eating | +0.37 | **0.016** |
| Q26 | I am always hungry so it is hard for me to stop eating before I finish the food on my plate. | Susceptibility to hunger | -0.37 | **0.016** |

Abbreviations: Q, question; T1, timepoint 1 (0 months); T3, timepoint 3 (12 months).

For comparisons, we used McNemar’s test of symmetry for dependent variables, and considered *p* < 0.05 statistically significant. Significant values are shown in bold.
