# Supplementary material for: Opposing patterns in eating behaviors following bariatric surgery versus lifestyle-induced weight loss
Source: PLoS One. 2026 Apr 27;21(4):e0346240. doi: 10.1371/journal.pone.0346240 (PMC13119899; doi:10.1371/journal.pone.0346240)
Supplement: S8 Table — Abbreviations: Q, question; T1, timepoint 1 (0 months); T3, timepoint 3 (12 months). For comparisons, we used McNemar’s test of symmetry for dependent variables and considered p < 0.05 statistically significant. Significant values are shown in bold. (DOCX) [file pone.0346240.s008.docx]

**Supplementary Table 4e. Most changed individual questions from DEBQ between baseline and 12 months in the lifestyle-induced weight loss group.**

| **Lifestyle** | **Dutch Eating Behavior Questionnaire** | | | |
| --- | --- | --- | --- | --- |
| Question |  | Behavioral trait | Δmean (T3-T1) | Symmetry test p-value |
| Q29 | If you walk past a snackbar or a I, do you have the desire to buy something delicious? | External eating | -0.89 | **0.008** |
| Q24 | If food tastes good to you, do you eat more than usual? | External eating | -0.67 | **0.008** |
| Q31 | Can you resist eating delicious foods? | External eating | +0.94 | **0.016** |
| Q28 | If you walk past the baker do you have the desire to buy something delicious? | External eating | -0.67 | **0.031** |
| Q30 | If you see others eating, do you also have the desire to eat? | External eating | -0.56 | **0.031** |
| Q3 | How often do you refuse food or drink offered because you are concemed about your weight? | Restrained eating | +0.68 | **0.037** |
| Q4 | Do you watch exactly what you eat? | Restrained eating | +1.00 | 0.052 |
| Q2 | Do you try to eat less at mealtimes than you would like to eat? | Restrained eating | +0.53 | 0.098 |
| Q27 | If you have something delicious to eat, do you eat it straight away? | External eating | -0.72 | 0.14 |
| Q19 | Do you have a desire to eat when things are going against you or when things have gone wrong? | Emotional eating | +0.22 | 0.14 |

Abbreviations: Q, question; T1, timepoint 1 (0 months); T3, timepoint 3 (12 months).

For comparisons, we used McNemar’s test of symmetry for dependent variables, and considered *p* < 0.05 statistically significant. Significant values are shown in bold.
