# Supplementary material for: Opposing patterns in eating behaviors following bariatric surgery versus lifestyle-induced weight loss
Source: PLoS One. 2026 Apr 27;21(4):e0346240. doi: 10.1371/journal.pone.0346240 (PMC13119899; doi:10.1371/journal.pone.0346240)
Supplement: S9 Table — Abbreviations: Q, question; T1, timepoint 1 (0 months); T3, timepoint 3 (12 months). For comparisons, we used McNemar’s test of symmetry for dependent variables and considered p < 0.05 statistically significant. Significant values are shown in bold. (DOCX) [file pone.0346240.s009.docx]

**Supplementary Table 4f. Most changed individual questions from BES between baseline and 12 months in the lifestyle-induced weight loss group.**

| **Lifestyle** | **Binge-Eating Scale** | | |
| --- | --- | --- | --- |
|  | Statements | Δmean (T3-T1) | Symmetry test p-value |
| Q9 | 1. My level of calorie intake does not go up very high or go down very low on a regular basis. | -0.88 | **0.016** |
|  | 2. Sometimes after I overeat, I will try to reduce my caloric intake to almost nothing to compensate for the excess calories I’ve eaten. |  |  |
|  | 3. I have a regular habit of overeating during the night. It seems that my routine is not to be hungry in the morning but overeat in the evening. |  |  |
|  | 4. In my adult years, I have had week-long periods where I practically starve myself. This follows periods when I overeat. It seems I live a life of either “feast or famine.” |  |  |
| Q8 | 1. I rarely eat so much food that I feel uncomfortably stuffed afterwards. | -0.53 | 0.055 |
|  | 2. Usually about once a month, I eat such a quantity of food, I end up feeling very stuffed. |  |  |
|  | 3. I have regular periods during the month when I eat large amounts of food, either at mealtime or at snacks. |  |  |
|  | 4. I eat so much food that I regularly feel quite uncomfortable after eating and sometimes a bit nauseous. |  |  |
| Q5 | 1. I’m usually physically hungry when I eat something. | -0.24 | 0.063 |
|  | 2. Occasionally, I eat something on impulse even though I really am not hungry. |  |  |
|  | 3. I have the regular habit of eating foods, that I might not really enjoy, to satisfy a hungry feeling even though physically, I don’t need the food. |  |  |
|  | 4. Even though I’m not physically hungry, 1 get a hungry feeling in my mouth that only seems to be satisfied when I eat a food, like a sandwich, that fills my mouth. Sometimes, when I eat the food to satisfy my mouth hunger, I then spit the food out so I won’t gain weight. |  |  |
| Q13 | 1. I eat three meals a day with only an occasional between meal snack. | -0.53 | 0.13 |
|  | 2. I eat 3 meals a day, but I also normally snack between meals. |  |  |
|  | 3. When I am snacking heavily, I get in the habit of skipping regular meals. |  |  |
|  | 4. There are regular periods when I seem to be continually eating, with no planned meals. |  |  |
| Q10 | 1. I usually am able to stop eating when I want to. I know when “enough is enough.” | -0.47 | 0.13 |
|  | 2. Every so often, I experience a compulsion to eat which I can’t seem to control. |  |  |
|  | 3. Frequently, I experience strong urges to eat which I seem unable to control, but at other times I can control my eating urges. |  |  |
|  | 4. I feel incapable of controlling urges to eat. I have a fear of not being able to stop eating voluntarily. |  |  |

Abbreviations: Q, question; T1, timepoint 1 (0 months); T3, timepoint 3 (12 months).

For comparisons, we used McNemar’s test of symmetry for dependent variables, and considered *p* < 0.05 statistically significant. Significant values are shown in bold.
